# Large choroidal melanoma presenting as neovascular glaucoma

**DOI:** 10.3205/oc000108

**Published:** 2019-05-31

**Authors:** Sabin Sahu, Tshering Wangchuk Bhutia, Varun Shrestha, Sanjay Kumar Singh, Lila Raj Puri

**Affiliations:** 1 Sagarmatha Choudhary Eye Hospital, Siraha, Nepal

**Keywords:** choroidal melanoma, iris neovascularisation, neovascular glaucoma

## Abstract

Choroidal melanoma is a relatively rare tumour with a poor prognosis, though it is the most common primary malignancy of the eye among adults. Choroidal melanoma has been reported to present as acute angle closure glaucoma, secondary glaucoma, chronic uveitis, cataract, and staphyloma. We report a case of a28-year-old male presenting with features of neovascular glaucoma in the right eye and having initially been treated with anti-glaucoma medications. However, ultrasonography revealed a mushroom-shaped, elevated, solid lesion with low to moderate internal reflectivity and regular internal structure suggestive of choroidal melanoma. Histopathological examination along with the immunohistochemistry studies of the lesion following enucleation of the eye confirmed the diagnosis of malignant choroidal melanoma. We highlight with this case that large choroidal melanoma may rarely present with features of neovascular glaucoma. The etiology of neovascular glaucoma should be investigated carefully and a potentially life-threatening intraocular tumour should be excluded, although it is a rare presentation.

## Introduction

Choroidal melanoma is a relatively rare tumour with a poor prognosis, though it is the most common primary malignancy of the eye among adults [1]. Patients usually present with decreased vision, visual field defects, pain, photophobia and floaters or may remain asymptomatic. Tumours are usually discovered during routine eye examination. Numerous benign and malignant lesions may mimic its ophthalmoscopic features [2]. We report a case of large choroidal melanoma presenting with features of neovascular glaucoma. 

## Case description

**Figure 1:**
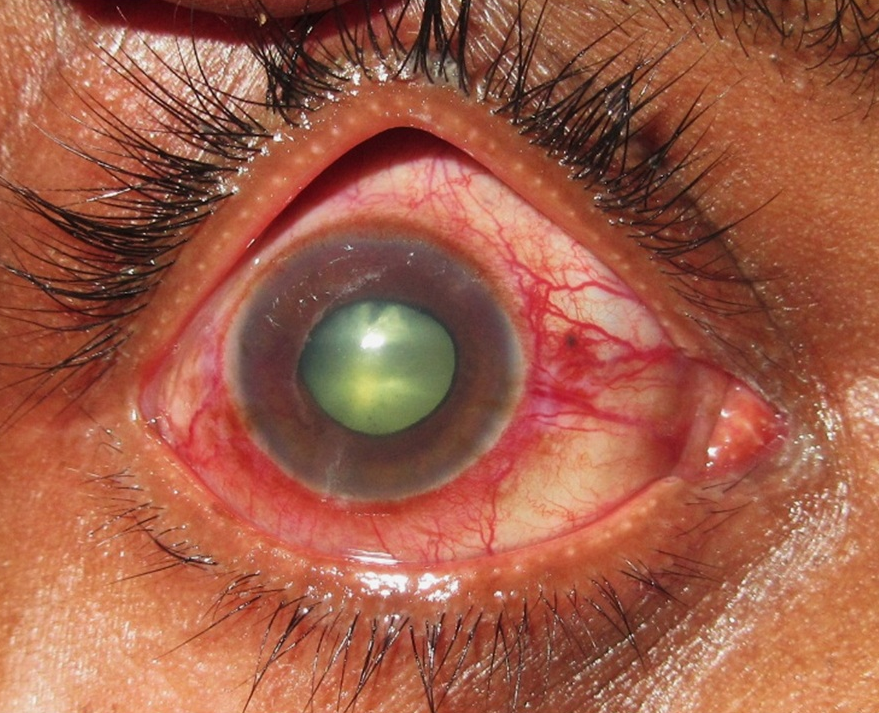
Anterior segment photo of the right eye showing conjunctival congestion, mid-dilated pupil, and iris neovascularization

A 28-year-old male with no known medical illness presented with a history of pain, redness and decreased vision in the right eye for the last 4 months. On examination, his visual acuity was no perception of light with intraocular pressure of 58 mmHg while he was on oral acetazolamide and topical beta blocker/alpha-2 agonist combination in the right eye. On slit lamp biomicroscopy examination, conjunctival congestion, corneal edema, mid-dilated pupil non-reacting to light, neovascularization of iris, and shallow anterior chamber with cellular reaction were noted in the right eye (Figure 1 (Fig. 1)). Fundus was not visible. 

**Figure 2:**
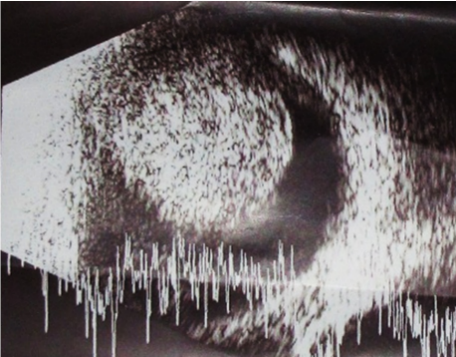
Ultrasonography showing a mushroom-shaped, elevated, solid lesion with regular internal structure and low to moderate internal reflectivity suggestive of choroidal melanoma

Gonioscopy examination with indentation revealed angle closure without any sign of neovascularization. The left eye showed a visual acuity of 20/20 with –0.75 DS correction, an intraocular pressure of 14 mmHg, a normal anterior chamber and normal disc and macula. Ultrasonography of the right eye revealed a mushroom-shaped, elevated, solid lesion in the superotemporal sector with a base diameter of approximately 15 mm with low to moderate internal reflectivity and regular internal structure suggestive of choroidal melanoma (Figure 2 (Fig. 2)). 

**Figure 3:**
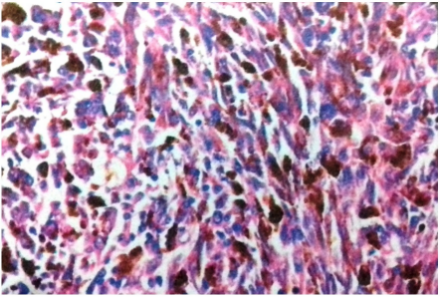
Histopathology demonstrating choroid tissue infiltration by a tumour arranged in sheets and fascicles with elongated spindle-shaped vesicular nuclei with prominent nucleoli and abundant melanin pigment (hematoxylin and eosin, original magnification 300x)

After systemic clinical evaluation and ruling out systemic involvement, the enucleation of the right eye was performed. The microscopic examination of the section showed choroid tissue infiltration by a tumour arranged in sheets and fascicles with elongated spindle-shaped vesicular nuclei with prominent nucleoli and abundant melanin pigment (Figure 3 (Fig. 3)). The tumour cells were positive for HMB45, S100 and Melan A. The histopathological examination along with the immunohistochemistry studies confirmed the diagnosis of malignant choroidal melanoma. The patient is under regular follow-up in outpatient care and has not shown any evidence of local or systemic relapse 1 year after the diagnosis and treatment. 

## Discussion

Uveal melanoma is the most common primary intraocular tumour in adults arising from iris, ciliary body, and most commonly the choroid [3]. It is not only vision threatening but also potentially fatal. Choroidal melanoma appears as a pigmented dome-shaped mass in fundus examination. The other clinical features may include vitreous hemorrhage, retinal detachment, choroidal detachment, ocular inflammation, and secondary glaucoma [4], [5]. It has also been reported to present as acute angle closure glaucoma [6], chronic uveitis [7], cataract, and staphyloma [8]. In our case, the patient presented with the features of neovascular glaucoma in the right eye and was being initially treated with anti-glaucoma medications elsewhere. The patient was referred to our centre for transscleral cyclophotocoagulation (TSCPC) in view of uncontrolled intraocular pressure and painful blind eye. However, detail clinical examination and ultrasonography revealed the intraocular mass suggestive of choroidal melanoma.

Secondary glaucoma may appear after diagnosis of choroidal melanoma or may be a presenting feature. The most common mechanism of increased intraocular pressure has been noted to be iris neovascularization in the case of choroidal melanomas [9]. Neovascularization is a result from ischemic necrosis of the tumour and hypoxic retinopathy mediated by vascular endothelial growth factor (VEGF) and other vasogenic factors like basic fibroblast growth factor (bFGF) present centrally within the tumour or adjacent to the areas of necrosis [10]. In a histological study of 223 eyes which underwent primary enucleation because of uveal melanoma rubeosis, iridis was found in 12.6% of the eyes [11].

Besides detailed clinical examination, ultrasonographic methods (A- and B-scan ultrasonography (USG), ultrasound biomicroscopy (UBM)), computerized tomography (CT) scan, fundus fluorescein angiography, indocyanin green angiography, optical coherence tomography, autofluorescence, fine needle aspiration biopsy, and cytogenetic analysis has been used for diagnosis and evaluation of uveal tumours [4]. In this case, the tumour was evaluated using A- and B-scan USG, which is easily accessible and has been proven as a very useful device for diagnosis of uveal tumours.

Enucleation was historically the mainstay of treatment for choroidal melanoma. Alternative treatment modalities in recent years include plaque radiotherapy, transscleral resection, elevation of tissue temperature, and transpupillary thermotherapy [1]. Although the enucleation rate has declined in recent decades with emergence of more conservative treatment options, enucleation remains a common treatment for large tumours or in cases with nil visual potential. In our case, the tumour was large and had nil visual prognosis, so enucleation was performed and the histopathological examination confirmed the diagnosis of malignant choroidal melanoma.

## Conclusion

Choroidal melanoma can rarely present with the features of neovascular glaucoma. Etiology of neovascular glaucoma should be investigated carefully and intraocular tumours should be kept as a differential diagnosis in suspicious conditions. Our case highlights the importance of meticulous clinical examination and thorough investigations to exclude intraocular tumour as a cause of secondary glaucoma, although it is a rare presentation.

## Notes

### Competing interests

The authors declare that they have no competing interests.
